# Factor XI Inhibitors for Prevention and Treatment of Venous Thromboembolism: A Review on the Rationale and Update on Current Evidence

**DOI:** 10.3389/fcvm.2022.903029

**Published:** 2022-05-12

**Authors:** Stephan Nopp, Daniel Kraemmer, Cihan Ay

**Affiliations:** Clinical Division of Hematology and Hemostaseology, Department of Medicine I, Medical University of Vienna, Vienna, Austria

**Keywords:** factor XI, anticoagulants, thrombosis, venous thromboembolism, hemorrhage, hemostasis

## Abstract

Although anticoagulation therapy has evolved from non-specific drugs (i.e., heparins and vitamin K antagonists) to agents that directly target specific coagulation factors (i.e., direct oral anticoagulants, argatroban, fondaparinux), thrombosis remains a leading cause of death worldwide. Direct oral anticoagulants (i.e., factor IIa- and factor Xa-inhibitors) now dominate clinical practice because of their favorable pharmacological profile and ease of use, particularly in venous thromboembolism (VTE) treatment and stroke prevention in atrial fibrillation. However, despite having a better safety profile than vitamin K antagonists, their bleeding risk is not insignificant. This is true for all currently available anticoagulants, and a high bleeding risk is considered a contraindication to anticoagulation. As a result, ongoing research focuses on developing future anticoagulants with an improved safety profile. Several promising approaches to reduce the bleeding risk involve targeting the intrinsic (or contact activation) pathway of coagulation, with the ultimate goal of preventing thrombosis without impairing hemostasis. Based on epidemiological data on hereditary factor deficiencies and preclinical studies factor XI (FXI) emerged as the most promising candidate target. In this review, we highlight unmet clinical needs of anticoagulation therapy, outlay the rationale and evidence for inhibiting FXI, discuss FXI inhibitors in current clinical trials, conduct an exploratory meta-analysis on their efficacy and safety, and provide an outlook on the potential clinical application of these novel anticoagulants.

## Introduction

### Evolution of Anticoagulation

Anticoagulation represents one of three forms of antithrombotic therapy, next to antiplatelet and thrombolytic therapy, and is the mainstay of prevention and treatment of venous thromboembolism (VTE), a disease entity covering pulmonary embolism and deep vein thrombosis. The landscape of anticoagulant drugs in use have shifted over the last decades from unspecific drugs with multiple pharmacological targets to agents specifically targeting a factor of the coagulation cascade. Long before the pharmacological mechanisms had been revealed, heparin and vitamin K-antagonists (VKA) were the first drugs in clinical use (1, 2). However, their narrow therapeutic window, unpredictable pharmacodynamic response, and serious, albeit rare, adverse events (e.g., heparin-induced thrombocytopenia, coumarin-induced skin necrosis) led to the search for safer anticoagulants.

The first evolutionary step in anticoagulant treatment involved the development of derivatives of heparin and coumarin. Low molecular weight heparin (LMWH) and coumarin derivatives such as warfarin and phenprocoumon were developed. Yet, these newer drugs still rely on a multi-targeted approach that impairs physiological hemostasis. Heparin is mediated by antithrombin and heparin cofactor II (3), and coumarins disrupt the synthesis of vitamin-K-dependent coagulation factors (4). The first selective inhibitor of a coagulation factor was fondaparinux, a synthetic heparin pentasaccharide inhibiting factor Xa (FXa) with a favorable pharmacokinetic profile, which, similar to LMWH, did not require coagulation monitoring (5). Development of anticoagulant therapy continued with the invention of selective inhibitors of thrombin (FIIa): bivalirudin and the small molecule argatroban. However, long-term application was still limited due to their need of parenteral administration. But the new era was finally ushered in with the invention of the direct oral anticoagulants (DOAC). Already in 2004, ximelagatran, a direct oral FIIa inhibitor, was developed but withdrawn from the market due to hepatotoxicity. Subsequently, the new class of DOAC (dabigatran, rivaroxaban, apixaban, edoxaban, and betrixaban), which selectively and reversibly inhibit FIIa or FXa in the common pathway of the coagulation cascade, was introduced in clinical practice in 2010, 2011, 2012, 2015, and 2017, respectively, and led to a revolution in anticoagulant therapy (6).

### The Current State of Anticoagulant Therapy

Predictable pharmacokinetics, ease of use, and a wide therapeutic window are the most important advantages of DOAC, leading to a quick uptake in prescriptions and broad use in clinical practice (6, 7). Today, DOAC are the most commonly used anticoagulants in the western world (8, 9), and two DOAC rank among the top 10 best-selling drugs worldwide (10). In a review on anticoagulants prescription trends in the primary care setting in England, the proportion of DOAC have increased from 9% in 2014 to 74% in 2019 (11). However, an analysis of electronic database searches suggests that VKA still remain the most popular anticoagulants worldwide (12). Possible reasons include limited access to DOAC in some parts of the world, considerably lower costs of VKA, which is particularly relevant in health systems without drug reimbursement, and physician and patient resistance to changing the standard of care. DOAC are most frequently prescribed for stroke prevention in patients with atrial fibrillation, followed by treatment of VTE and prevention of its recurrence (8). In VTE patients, DOAC are now recommended as the first choice for anticoagulant therapy (13–15). Compared to VKA, they have proven to be of similar efficacy (16–21) and are associated with a lower bleeding risk, particularly with respect to intracranial hemorrhage (22). However, higher rates of gastrointestinal bleeding have been reported for dabigatran, rivaroxaban, and edoxaban in special patient populations including patients with atrial fibrillation or cancer (23, 24). To mitigate bleeding risk, reduced-dose DOAC regimens are used in patients with specific dose adjustment criteria and for long-term prevention of VTE (i.e., for rivaroxaban and apixaban) (25). Moreover, specific reversal agents have been developed, and in 2015 idaruzimuab for dabigatran and in 2018 andexanet alfa for reversal of the FXa inhibitors apixaban and rivaroxaban have been approved and introduced for use in clinical practice (26, 27). Nevertheless, bleeding remains the most frequent complication of anticoagulation. Fear of bleeding contributes to underuse and underdosing of eligible patients, and a high risk of bleeding is the most important contraindication to long-term anticoagulation (28, 29).

### Unmet Clinical Needs

Patients in need of a better anticoagulation strategy can be divided into two groups. The first group represents patients with an increased risk of bleeding (e.g., patients with kidney impairment, prior history of bleedings, cancer, or the elderly). Notably, bleeding risk assessment is challenging and no single approach has been proved to be consistently superior (30). Thus, the decision to initiate or continue anticoagulation remains at the discretion of the treating physician based on an individual assessment of risk of thrombosis and bleeding. Accordingly, anticoagulants with an improved safety profile could serve as a novel and important treatment option in patients in whom the risk of bleeding is higher than the risk of thrombosis. A second group that might benefit from new treatment options includes patients in whom the efficacy of DOAC has been tested but has shown to be inferior to VKA, or in whom there is insufficient evidence to support the use of DOAC, such as patients whose cardiovascular system is exposed to artificial surfaces. In patients with mechanical heart valves, dabigatran was inferior to VKA (31), and randomized trials of other DOAC against VKA are lacking. In dialysis patients, where the risk of bleeding is exceptionally high (32), the benefit-to-risk ratio of oral anticoagulants, in general, is still debated as observational studies report excess bleeding rates with VKA and DOAC (33, 34). In patients with left ventricular assist devices or extracorporeal membrane oxygenation, DOAC have not been assessed in randomized-controlled trials. Further, VKA are recommended over DOAC in patients with antiphospholipid syndrome as two randomized trials were prematurely terminated due to excess thromboembolic events in the DOAC compared to the VKA treatment arm (35, 36). Taken together, a significant proportion of patients need safer anticoagulants or anticoagulants designed to prevent and treat thrombosis triggered by artificial surfaces.

### Thrombogenesis—Revisiting the Coagulation Cascade

The formation of a blood clot, i.e., thrombogenesis, is the combined result of increased coagulation, hemodynamic changes in the circulation, and vascular endothelial injury (37). Anticoagulation is effective to prevent and treat thrombosis but also interferes with hemostasis, and leads to increased bleeding risk (38).

**FIGURE 1:**
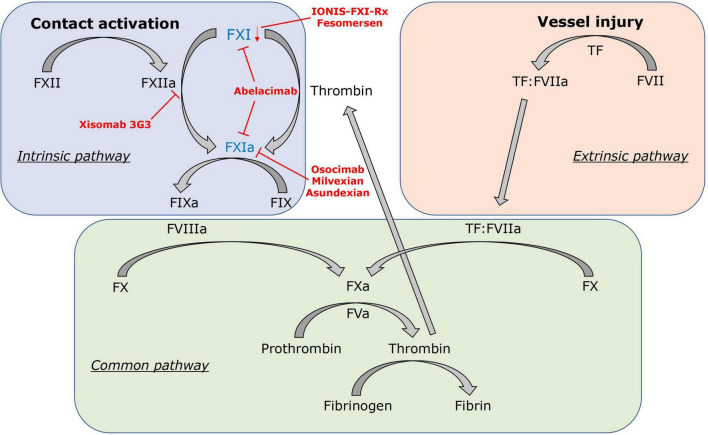
Factor XI in the clotting cascade. FXI is activated by the contact activation system *via* FXIIa, but also contact factor independent *via* the positive feedback loop of thrombin (FIIa). In red, FXI inhibitors and their mechanisms of action are displayed: IONIS-FXI-Rx and fesomersen reduce FXI messenger RNA expression in the liver. Abelacimab inhibits FXI and FXIa. Osocimab, milvexian, and asundexian inhibit FXIa. Xisomab 3G3 blocks FXIIa-mediated FXI activation without inhibiting FXI activation by thrombin.

Different models to explain the coagulation process *in vitro* and *in vivo* have been proposed. The classical clotting cascade model consists of the intrinsic and extrinsic pathways that converge in the common pathway (FX, FV, FII) (Figure 1) (39, 40). The extrinsic coagulation pathway [tissue factor (TF), FVII], which is essential for fibrin formation at a site of vascular injury, is initiated by exposure of TF to blood and formation of the TF:FVIIa complex (41). The intrinsic coagulation pathway (FXII, FXI, FIX, FVIII) is initiated by contact activation mediated by FXII when blood interacts with certain negatively charged surfaces. This strict separation of both pathways is based on *in vitro* observations. In the last two decades, the cell-based coagulation model, which interconnects both pathways, gained acceptance and better reflects the hemostatic processes *in vivo* (42–44). Based on this theory, coagulation or pathological thrombosis is initiated by the exposure of TF from TF-bearing cells (representing the extrinsic pathway). Importantly, large amounts of thrombin are needed thereafter for both thrombosis and hemostasis, which are generated by subsequent activation of the intrinsic pathway (44). *In vivo*, activation of the intrinsic pathway through the contact system is initiated by FXII activation *via* exposure to polyanionic compounds originating from injured cells, activated platelets, or pathogens (45). However, in this cell-based model, activation of the intrinsic pathway also occurs independently of the contact system. FXI is activated by FIIa generated from the extrinsic pathway and FIX by the TF:FVIIa complex (extrinsic FXase) (46). Subsequently, FXIa and FIXa contribute to the amplification of thrombin production *via* the “truncated” intrinsic pathway.

Modern coagulation theory hypothesizes that the mechanisms of pathological thrombosis and physiological hemostasis are distinguishable, but all currently available anticoagulants in clinical use either target coagulation FII or FX, both essential factors for physiological hemostasis. Inhibition of either one of the two factors interrupts the common pathway and disrupts the positive feedback activation of FV, FVIII, and FXI. Upstream the common pathway, initiation of hemostasis mainly depends on the TF-mediated extrinsic pathway, and deficiency or increased activity of this pathway leads to bleeding or thrombosis. The intrinsic pathway, which is activated by the contact activation system and resembles an interface between inflammation, coagulation, and innate immunity, however, contributes weakly to hemostasis but is closely associated with pathological thrombosis (47–50). In the presence of excessive TF, thrombin generation is likely sufficient for hemostasis without a necessary involvement of the intrinsic pathway. But in the presence of limited TF, activation of FX through the intrinsic FXase is required for rapid and sufficient production of thrombin as seen in various pathological processes (e.g., immunothrombosis, atherothrombosis) (51, 52). Thus, revisiting the intrinsic pathway with its contact activation system in the effort to inhibit the coagulation cascade upstream of FX is a noteworthy approach in the desire to prevent thrombosis without the cost of impairing hemostatic functions.

### The Intrinsic Pathway, Contact System, and Rationale for Targeting Factor XI

Anticoagulant-related bleeding might be avoided if coagulation factors that are more important for pathological thrombosis than for physiological hemostasis are targeted selectively (53). Accordingly, the detrimental role of contact system-dependent coagulation activation in thrombus formation proposes the intrinsic coagulation pathway as a potential target for novel anticoagulants. The intrinsic pathway begins with activation through the contact system, which consists of FXII, prekallikrein (PK), and a cofactor high-molecular-weight kininogen (HK) (54). This contact activation is triggered when plasma encounters certain negatively charged macromolecules, leading to the activation of FXII. Subsequently, FXI and FIX get activated. Therefore, factors of the intrinsic pathway represent promising candidate targets for antithrombotic therapy (55–57). Notably, FXI research has proceeded furthest in clinical trials having shown the most promising evidence from epidemiological studies and animal models.

FXI, discovered in 1953, is a serine protease that originated from duplication of the prekalikrein gene. It can be activated by FXIIa through the contact activation pathway but also acts independently of contact activation when activated by thrombin (FIIa) *via* the positive feedback loop (Figure 1). FXI is considered essential for thrombus growth and stabilization but might have a subsidiary role in physiological hemostasis (58, 59). In epidemiological and Mendelian randomization studies, elevated FXI has been linked to stroke and VTE (60–62). Furthermore, studies on lower levels of FXI or FXI-deficiency showed that decreased FXI levels were associated with a lower risk of VTE and cardiovascular events (63–66). However, patients with FXI-deficiency also appear to be at higher risk for post-traumatic bleeding, whereas patients deficient in contact factors (FXII, PK, HK) have no apparent bleeding diathesis (67). Such findings could be viewed as disproof to the theory of separating thrombosis from hemostasis by FXI inhibition. However, FXI activity levels only poorly correlate with bleeding risk (67), and congenital FXI deficiency is rarely associated with spontaneous bleeding (68). It has been reported that bleeding most likely occurs in individuals with FXI levels lower than 20% (69, 70). Thus, a threshold of 20% of FXI activity levels is typically used to define severe FXI deficiency (66, 71, 72) and moderate inhibition of FXI at or above this threshold might pose a solution to the coagulation dilemma. This hypothesis is further supported by findings from large genome-wide association studies. Genetic disposition to lower FXI levels was associated with a reduced risk for VTE and ischemic stroke but without increased risk for major bleeding (73). Evidence to inhibit FXI is further supported by animal studies. In animal models of thrombosis, FXI-deficient mice were protected from occlusive thrombosis in the vena cava (74), as well as from thrombosis in the arterial system (75). Interestingly, in an animal model of tail or surgical bleeding, mice with FXI deficiency displayed a normal bleeding phenotype, whereas a saphenous vein injury model revealed reduced hemostasis (76). Furthermore, inhibition of FXI in rats was effective in preventing venous thrombosis without increased bleeding in one study and minimal bleeding risk in another (77, 78). Various other studies have also shown similar findings for FXII (79, 80), but preclinical studies on inhibition of FXI and FXII in non-human primates suggested a greater antithrombotic effect when targeting FXI (81–84), providing the basis for FXI as the primary target for a new generation of anticoagulants in clinical trials.

### Clinical Trials of Factor XI Inhibition

Early clinical evaluation of novel anticoagulants usually starts with dose-finding studies in patients undergoing elective knee arthroplasty. This is considered a time-efficient way due to the high number of VTE events after surgery and the objective and efficient evaluation of drug efficacy using venography to determine the incidence of deep vein thrombosis of the lower extremity. Four of these studies on FXI inhibition have already been completed, in which clinically relevant bleeding was determined as the primary safety endpoint by combining major bleeding and clinically relevant non-major bleeding (85–88). The second type of FXI inhibitor studies focused on patients with end-stage kidney disease on hemodialysis (89, 90). High risk of bleeding and thrombosis provoked by the extracorporeal device makes them the prime target for potentially safer anticoagulants inhibiting the intrinsic pathway. In the following, all FXI inhibitors that have already completed phase II in clinical trial evaluation are discussed in more detail ordered by appearance of publication (Table 1). In Figure 1, FXI inhibitors and their mechanisms of action are displayed.

**TABLE 1:** Overview of factor XI inhibitors in clinical trials.

Drug	Type	Mechanism	Administration route	Studies (NCT)	Population (N)	Comparator	Status
IONIS-FXI_Rx_	Antisense oligonucleotide of FXI	Inhibits FXI messenger RNA	Subcutaneous (weekly)	NCT01713361 NCT02553889 NCT03358030	TKA (300) ESKD (49) ESKD (200)	Enoxaparin Placebo Placebo	Published Published Completed

Osocimab	Monoclonal antibody to FXIa	Binds and inhibits FXIa	Intravenous, subcutaneous (monthly)	NCT03276143 NCT04523220	TKA (813) ESKD (686)	Enoxaparin/Apixaban Placebo	Published Ongoing

Abelacimab	Monoclonal antibody to FXI/FXIa	Binds and inhibits FXI and FXIa	Subcutaneous (monthly)	EudraCT 2019-003756-37 NCT04755283 NCT05171049 NCT05171075	TKA (412) AF (1,200) CAT (1,655) CAT (1,020)	Enoxaparin Rivaroxaban Apixaban Dalteparin	Published Ongoing Ongoing Ongoing

Milvexian	Small molecule inhibitor of FXIa	Binds and inhibits FXIa	Oral (daily)	NCT03891524 NCT03766581	TKA (1,242) Stroke (2,366)	Enoxaparin Placebo	Published Ongoing

Xisomab 3G3	Monoclonal antibody to FXI	Binds FXI and blocks activation by FXIIa	Intravenous (single dose)	NCT03612856 NCT04465760	ESKD (24) CRT (50)	Placebo None	Published Ongoing

Fesomersen	Antisense oligonucleotide of FXI	Inhibits FXI messenger RNA	Subcutaneous (weekly)	NCT04534114	ESKD (305)	Placebo	Ongoing

Asundexian	Small molecule inhibitor of FXIa	Binds and inhibits FXIa	Oral (daily)	NCT04218266 NCT04304534 NCT04304508	AF (753) AMI (1,592) Stroke (1,790)	Apixaban Placebo Placebo	Published Completed Ongoing

*AF, atrial fibrillation; CAT, cancer-associated thrombosis; CRT, catheter-related thrombosis in cancer patients; ESKD, end-stage kidney disease; TKA, total knee arthroplasty.*

**IONIS-FXI_*RX*_/FXI-ASO,** an antisense oligonucleotide, was the first agent investigated in a phase II trial in patients undergoing total knee arthroplasty. Antisense oligonucleotides are attractive novel therapeutics that are highly bound to plasma proteins limiting glomerular filtration and urinary excretion, have no known drug-to-drug interaction and require subcutaneous application. IONIS-FXI_*Rx*_ blocks the hepatic synthesis of FXI by specifically reducing FXI messenger RNA levels in the liver. As such, it takes several weeks to lower FXI to therapeutic levels, and thus the restoration to normal levels is also delayed for several weeks after treatment discontinuation (81). In a randomized controlled trial of 300 patients, efficacy and safety of IONIS-FXI_*Rx*_ at doses of 100, 200, and 300 mg were evaluated and compared to enoxaparin 40 mg once daily (85). Treatment with IONIS-FXI_*Rx*_ was initiated 36 days before surgery and patients received 9 subcutaneous doses over a total of 39 days. Shortly after the start of the trial, the 100 mg dose regimen was discontinued due to insufficient reduction of FXI levels and replaced by the 300 mg dose regimen. In this study, the 200 mg dose regimen proved non-inferior and the 300 mg dose regimen superior compared to enoxaparin in preventing VTE. In all knee arthroplasty studies, VTE was the primary endpoint as assessed by venography. The rates of VTE were 27% in the 200 mg arm, 4% in the 300 mg arm, and 30% in the enoxaparin treatment group. Bleeding rates did not statistically differ with lower rates in IONIS-FXI_*Rx*_ groups (3% in both treatment groups vs. 8% in enoxaparin arm). Serious adverse events occurred in the IONIS-FXI_*Rx*_ treatment groups but were deemed unlikely to be associated with the drug by the investigators.

The second phase II study with this agent was conducted in 49 patients with end-stage kidney disease (89). In the first part of the study, 6 patients received 300 mg of IONIS-FXI_*Rx*_ before and after hemodialysis. In the second part, 43 patients received either 200 mg, 300 mg, or placebo for 12 weeks in a randomized fashion. The main goal was to investigate pharmacokinetics, -dynamics, and adverse events. Notably, no pharmacokinetic differences were observed between injections pre and post hemodialysis. Further, no drug accumulation and no drug-related serious adverse events were noted. Importantly, the study was not powered to estimate efficacy and safety, but predefined comparisons of the clot burden of the hemodialysis circuit suggested a substantial reduction (up to -29%) in severe clotting events after treatment onset of IONIS-FXI_*Rx*_. Clinically relevant bleeding occurred in one patient in the placebo and in one patient in the IONIS-FXI_*Rx*_ group, but minor bleeding was numerically more frequent in patients with the study drug (23 vs. 8%). Another subsequent study in end-stage kidney disease patients has already been completed but not yet published (NCT03358030). Further studies with the ligand-conjugated version of IONIS-FXI_*Rx*_, termed fesomersen or IONIS-FXI-L_*Rx*_ are ongoing (NCT04534114).

**Osocimab**, is a long-acting, fully human monoclonal antibody with a time to maximum plasma concentration of 1–4 h and a half-life of 30–44 days after intravenous application. It binds adjacent to the active site of FXIa and prevents it from activating FIX. In this first trial on FXIa inhibition, efficacy and safety of 4 different doses (0.3, 0.6, 1.2, 1.8 mg/kg) administered pre- or postoperatively *via* a single, 60-min, intravenous infusion were compared to 40 mg enoxaparin once daily or 2.5 mg apixaban twice daily in 813 patients who underwent total knee replacement (86). Initially, three dose levels of osocimab administered postoperatively were evaluated, but during the ongoing study and without any safety concerns being raised, a higher postoperative dose (1.8 mg/kg) and two preoperative doses (0.3, 1.8 mg/kg) were initiated as prespecified. In the per-protocol analysis, which consisted of only 74% of randomized patients due to missing data from venography, non-inferiority to enoxaparin was shown for the 3 higher doses of osocimab (0.6, 1.2, 1.8 mg/kg). Superiority was shown for the highest dose given preoperatively, while non-inferiority could not be attributed to 0.3 mg/kg osocimab. VTE rates in the treatment groups ranged from 23.7 to 15.7% with postoperative osocimab and 29.9–11.3% with preoperative osocimab compared to 26.3% in the enoxaparin group and 14.5% in the apixaban group. Only one major bleeding event occurred in the study in a patient with a preoperative dose of 1.8 mg/kg. In summary, bleeding rates in the osocimab treatment groups were lower compared to the control group and ranged between 1% with 0.3 mg/kg and 4.7% with 1.8 mg/kg osocimab compared to 5.9% in the enoxaparin arm. In the apixaban group, only 2% of patients experienced clinically relevant bleeding. Serious adverse events were similarly distributed across groups, but higher rates of hypersensitivity and infusion-related reactions were reported for osocimab. However, none resulted in discontinuation of the study drug. A study with anticipated 600 patients with end-stage kidney disease on hemodialysis is underway (NCT04523220).

**Abelacimab** is a monoclonal antibody that binds to the catalytic domain of FXI and locks it in its zymogen (inactive precursor) conformation, thereby preventing its activation by FXIIa or thrombin (FIIa) (91). Three different dosing regimens of abelacimab (30, 75, 150 mg) were tested and randomized against 40 mg of enoxaparin in patients undergoing knee replacement (87). Given the immediate method of action, the monoclonal antibody was administered in a single, intravenous infusion 4–8 h after surgery. After 412 from the anticipated 600 patients were included, the trial was stopped early. This decision was driven by slowed recruitment because of the Covid-19 pandemic. Nevertheless, power was sufficient to show non-inferiority to enoxaparin for all three regimens of abelacimab, while the 75 and 150 mg doses even met criteria for superiority. Rates for the primary efficacy endpoint VTE were 13, 5, 4, and 22% for 30, 75, 150 mg of abelacimab, and 40 mg of enoxaparin, respectively. No clinically relevant bleeding occurred in the enoxaparin arm and the abelacimab treatment group with the highest dose. In the two remaining groups, 2% experienced clinically relevant non-major bleeding. In total, 5 serious adverse events were reported in the abelacimab groups including wound infections, ileus, and ovarian cyst torsion. A randomized study with rivaroxaban as comparator evaluating safety but not efficacy of abelacimab is currently ongoing in 1,200 patients with atrial fibrillation (NCT04755283), and two phase III trials on cancer-associated thrombosis with the aim to enroll 1,655 and 1,020 patients, respectively, have recently been initiated (NCT05171049, NCT05171075).

**Milvexian,** a small molecule with oral bioavailability and a half-life of approximately 12 h, is a selective, reversible, direct inhibitor of FXIa. It has been studied in the largest phase II study so far, which included 1,242 patients undergoing knee arthroplasty to compare 7 different postoperative regimens of milvexian to enoxaparin for the efficacy of preventing VTE and the safety with regard to bleeding events and serious adverse events (88). Both predefined proof of efficacy criteria (i.e., a significant dose-response relationship trend and a VTE incidence that was significantly lower than 30% with the pooled twice-daily milvexian regimens) were met. Furthermore, 4 of the 7 dose regimens (50, 100, 200 mg twice daily, and 200 mg once daily) showed superiority compared to 40 mg of enoxaparin. Serious adverse events were rare and equally distributed across all treatment groups and no major bleeding event occurred in the milvexian treatment groups. Clinically relevant non-major bleeding was below or at 1% with no dose-response relationship seen across the seven different regimens. Similar to the previous studies mentioned, milvexian increased the aPTT ratio in a dose-dependent manner, while enoxaparin did not. A second study in the patients with ischemic stroke is still ongoing (NCT03766581).

**Xisomab 3G3/AB023** is a recombinant antibody that binds to FXI and selectively reduces its activation by FXIIa without attenuating the feedback mechanism by thrombin (FIIa) on FXI. Due to this unique mechanism, it rather acts as a FXIIa inhibitor and might be categorized as such. Interestingly, the elimination half-life of xisomab 3G3 increases with dose. At a dose of 0.1 mg/kg, a half-life of 1.3 h was measured; at administration of 5 mg/kg, the half-life increased to 121 h (92). This phenomenon of a short half-life at lower doses is likely due to the rapid binding of a large proportion of free xisomab 3G3 to FXI (93). Xisomab 3G3 was tested in a randomized controlled trial of 24 patients with end-stage kidney disease in need of heparin-free hemodialysis (90). Patients were randomized to 0.25, 0.5 mg/kg xisomab 3G3, or placebo injected as a single bolus into the dialysis line proximal to the dialyzer at the start of hemodialysis. The study’s primary aim was to assure safety with regard to bleeding events in this high-risk population. There were no clinically relevant bleeding events while patients were on the study drug, and the time to hemostasis at vascular access sites (i.e., the duration of pressure applied to the access site until bleeding stopped) was unchanged after xisomab 3G3 administration compared to before. One major bleeding event occurred 32 days after drug discontinuation but was deemed unrelated to the study drug by the study investigator. Results on efficacy were underpowered and exploratory but suggested a reduction of occlusive events requiring circuit exchange and lower levels of C-reactive protein and thrombin-antithrombin complexes in patients receiving xisomab 3G3. A subsequent study for the prevention of catheter-associated thrombosis in patients with cancer (NCT04465760) is currently ongoing to further investigate a proof of concept in patients exposed to artificial surfaces.

**Asundexian** is a small molecule direct inhibitor of FXIa with oral bioavailability and a terminal half-life of approximately 17 h. Asundexian is renally eliminated to less than 15% and has no clinically relevant interaction with cytochrome P450 (CYP) 3A4 (94). The safety of 2 different dose regimens (20 and 50 mg once daily) was studied in a randomized, double-blind, phase II study in 753 patients with atrial fibrillation and moderate-to-high risk for stroke and bleeding (95). Apixaban, the DOAC believed to have the lowest bleeding risk, was used as a control. Patients were treated for a period of 12 weeks and followed up for safety outcomes. The primary endpoint was the composite of major bleeding and clinically relevant non-major bleeding as defined by the International Society on Thrombosis and Hemostasis (ISTH). After 4 weeks of treatment, asundexian 20 and 50 mg at trough levels resulted in an 81 and 92% reduction in FXIa activity levels, respectively. In total, 10 clinically relevant non-major bleeding and no major bleeding occurred during the study period. The bleeding rates were 1.2, 0.4, and 2.4% in the asundexian 20 mg, asundexian 50 mg, and apixaban treatment groups, respectively, which showed a significant reduction in bleeding rates at a prespecified 90% confidence interval for the 50 mg regimen and in pooled analysis of both asundexian treatment groups. The rates of any adverse event and the rates of adverse events leading to drug discontinuation were similar across treatment groups, but 2 and 1 patients experienced ischemic stroke in the asundexian 20 and 50 mg arm, respectively, while none suffered from stroke in the apixaban group. In summary, asundexian was safer than apixaban with regard to bleeding events, but efficacy needs to be determined in phase III trials. A second phase II trial in 1,592 patients with recent myocardial infarction has already been completed but not published yet (NCT04304534) and one more study in patients with recent non-cardioembolic stroke is currently ongoing (NCT04304508).

### Meta-Analysis of Phase II Studies With Factor XI Inhibitors

**FIGURE 2:**
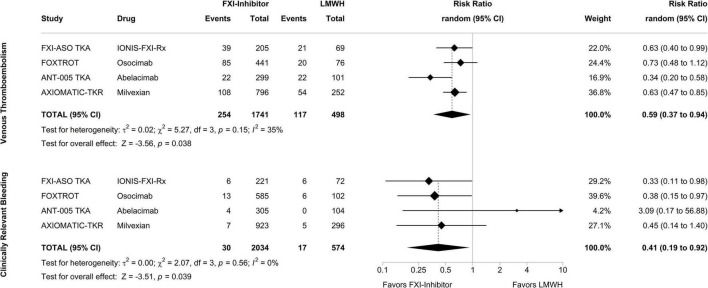
Meta-analysis of phase II studies assessing the efficacy and safety of factor XI inhibitors compared to enoxaparin for prevention of venous thromboembolism in patients undergoing total knee arthroplasty. After combining the treatment arms of the study drug in each phase II study, factor XI inhibitors were compared to enoxaparin in a random-effects model with the amount of heterogeneity (τ^2^) estimated using the restricted maximum-likelihood estimator. The analysis was carried out separately for the primary efficacy endpoint, venous thromboembolism, and the primary safety endpoint, clinically relevant bleeding, with outcomes depicted as risk ratios. Tests and 95% confidence intervals (95% CI) were calculated using the Knapp and Hartung method. The quantitative synthesis was conducted using R (version 4.1.3) and the metafor package (version 3.0.2).

To explore the efficacy and safety of FXI inhibitors in completed clinical trials, we performed an exploratory meta-analysis of all phase II studies investigating inhibitors of FXI in patients undergoing total knee arthroplasty. The specified endpoints were confirmed VTE for the efficacy analysis and clinically relevant bleeding (the composite of major bleeding and clinically relevant non-major bleeding) for the safety analysis. Figure 2 displays forest plots summarizing the occurrence of VTE and clinically relevant bleeding events in the 4 phase II studies, respectively, with treatment arms of each study pooled and compared against the enoxaparin comparator group. In a random-effects meta-analysis, treatment with a FXI inhibitor, when compared to 40 mg of enoxaparin, was associated with a decreased risk of VTE by a risk ratio of 0.59 (95% CI, 0.37–0.94; *p* = 0.038). Strikingly, the number of bleeding events in patients treated with FXI inhibitors was also reduced: the risk ratio to develop clinically relevant bleeding in patients treated with a FXI inhibitor compared to enoxaparin was 0.41 (95% CI, 0.19–0.92; *p* = 0.039).

## Conclusion and Potential Indications

Patients with an elevated risk of bleeding and patients whose blood is exposed to artificial surfaces require novel therapeutic concepts to attenuate the risk of thrombosis without increasing the risk of bleeding. Currently, all anticoagulant strategies target factors of the common pathway of the coagulation cascade, while a more upstream inhibition could be a promising treatment approach in the desire to prevent thrombosis without impairing hemostasis. It is of note that hemostasis strongly depends on the extrinsic pathway, whereas the intrinsic pathway seems less relevant for physiological processes but contributes to thrombosis. As a result, research is currently focused on inhibiting the intrinsic pathway, with FXI emerging as the most promising candidate. Six different inhibitors of FXI successfully completed phase II trials in patients undergoing total knee arthroplasty, patients suffering from end-stage kidney disease, and patients with AF. A new term summarizing these new anticoagulants targeting the intrinsic pathway has not been defined yet. Taken together, all inhibitors under investigation in knee arthroplasty studies proved to be at least non-inferior to LMWH in preventing VTE and highlighted the role of FXI in the pathogenesis of post-surgical thrombosis. This was somewhat surprising as knee arthroplasty is considered a prime example of extrinsic coagulation cascade activation due to a strong TF trigger. Pooled study results suggested a 41% reduction of VTE events with FXI inhibitors compared to LMWH (Figure 2). Of note, the meta-analysis on the safety outcome (i.e., clinically relevant bleeding) also suggested a 59% reduction of risk of bleeding in patients treated with a FXI inhibitor compared to enoxaparin. The currently available studies in patients with end-stage kidney disease and patients with atrial fibrillation were not powered to determine efficacy of the novel drugs but were reassuring in terms of safety, pharmacodynamics, and –kinetics to progress to phase III studies. The findings from the knee replacement studies are limited by the exclusion of patients with elevated risk of bleeding, a potentially important patient population eligible for FXI inhibitors. Moreover, the vast majority of recorded endpoints were asymptomatic distal deep vein thrombosis, events with less clinical relevance. Furthermore, there were signs of higher bleeding risk with higher doses in some FXI inhibitors indicating impaired hemostatic functions. Nevertheless, the combined study results leave hope to reach the promised land in anticoagulation therapy, the separation from thrombosis and hemostasis, a goal which would be particularly important for patients in need of safer anticoagulants or anticoagulants with better efficacy in conditions involving artificial surfaces (medical devices, extracorporeal circuits). But given the triumph of the DOAC, FXI inhibitors will face an uphill battle to establish their role in clinical practice. Potential indications will heavily rely on the different pharmacokinetics and -dynamics of each inhibitor. Agents that require weekly subcutaneous administration and which are not renally cleared might be good candidates for patients on hemodialysis (e.g., IONIS-FXI_*Rx*_, fesomersen). FXI inhibitors administered monthly could be given to patients with regular doctoral visits such as patients with cancer-associated thrombosis (e.g., abelacimab). FXI inhibitors with oral bioavailability might be more suitable for long-term VTE or stroke prevention (e.g., milvexian, asundexian), while drugs with short half-lives might be more appropriate for periinterventional use such as placement of catheter or extracorporeal circuits (e.g., low-dose xisomab 3G3). Potential differences in efficacy and safety between FXI inhibitors might be interpretable from the first published trials, but further trials will determine which inhibitor could be used in high-risk thrombotic situations, in patients with extremely high bleeding risk, or with concomitant use of antiplatelet agents. Lastly, given the signs of disrupted hemostasis for at least some of the FXI inhibitors at higher doses, additional development of reversal agents particularly for drugs with long half-lives, may be a relevant issue to consider.

## Author Contributions

SN had the idea for the review, performed literature research, and wrote the first draft of the manuscript. DK performed data analysis and critically revised the work. CA supervised the work and critically revised the manuscript. All authors contributed to the article and approved the submitted version.

## Conflict of Interest

CA declares personal fees for lectures and/or participation in advisory boards from Bayer, BMS, Pfizer, Daiichi-Sankyo, Sanofi. The remaining authors declare that the research was conducted in the absence of any commercial or financial relationships that could be construed as a potential conflict of interest.

## Publisher’s Note

All claims expressed in this article are solely those of the authors and do not necessarily represent those of their affiliated organizations, or those of the publisher, the editors and the reviewers. Any product that may be evaluated in this article, or claim that may be made by its manufacturer, is not guaranteed or endorsed by the publisher.

## References

[1] Copeland CE Six CK. A tale of two anticoagulants: warfarin and heparin. *J Surg Educ.* (2009) 66:176–81. 10.1016/j.jsurg.2009.03.035 19712919

[2] Weitz JI Harenberg J. New developments in anticoagulants: past, present and future. *Thromb Haemost.* (2017) 117:1283–8. 10.1160/TH16-10-0807 28594426

[3] Li W Johnson DJ Esmon CT Huntington JA. Structure of the antithrombin-thrombin-heparin ternary complex reveals the antithrombotic mechanism of heparin. *Nat Struct Mol Biol.* (2004) 11:857–62. 10.1038/nsmb811 15311269

[4] Vermeer C Schurgers LJ. A comprehensive review of vitamin K and vitamin K antagonists. *Hematol Oncol Clin North Am.* (2000) 14:339–53. 10.1016/s0889-8588(05)70137-4 10806559

[5] Torri G Naggi A. Heparin centenary–an ever-young life-saving drug. *Int J Cardiol.* (2016) 212(Suppl. 1):S1–4. 10.1016/S0167-5273(16)12001-7 27264864

[6] Franco Moreno AI Martín Díaz RM García Navarro MJ. Direct oral anticoagulants: an update. *Med Clin.* (2018) 151:198–206. 10.1016/j.medcli.2017.11.042 29295790

[7] Ilomäki J Fanning L Keen C Sluggett JK Page AT Korhonen MJ Trends and predictors of oral anticoagulant use in people with Alzheimer’s disease and the general population in Australia. *J Alzheimers Dis.* (2019) 70:733–45. 10.3233/JAD-190094 31256129

[8] Loo SY Dell’Aniello S Huiart L Renoux C. Trends in the prescription of novel oral anticoagulants in UK primary care. *Br J Clin Pharmacol.* (2017) 83:2096–106. 10.1111/bcp.13299 28390065 PMC5555878

[9] Birkinshaw A Fry CH Fluck D Sharma P Han TS. Changing trends in the use of novel oral anticoagulants and warfarin for treating non-valvular atrial fibrillation. *JRSM Cardiovasc Dis.* (2020) 9:2048004020915406. 10.1177/2048004020915406 32284860 PMC7119231

[10] PharmaDigiCoach. *Biggest Blockbuster Drugs of 2020 : Drug Sales and Beyond.* (2021). Available online at: https://www.pharmadigicoach.com/top-selling-drugs-by-2020-sales/ (accessed September 19, 2021).

[11] Ho KH van Hove M Leng G. Trends in anticoagulant prescribing: a review of local policies in English primary care. *BMC Health Serv Res.* (2020) 20:279. 10.1186/s12913-020-5058-1 32245380 PMC7126454

[12] Lippi G Mattiuzzi C Adcock D Favaloro EJ. Oral anticoagulants around the world: an updated state-of-the art analysis. *Ann Blood.* (2018) 3:49. 10.21037/aob.2018.12.04

[13] Kearon C Akl EA Ornelas J Blaivas A Jimenez D Bounameaux H Antithrombotic therapy for VTE disease: chest guideline and expert panel report. *Chest.* (2016) 149:315–52. 26867832 10.1016/j.chest.2015.11.026

[14] Konstantinides SV Meyer G Becattini C Bueno H Geersing GJ Harjola VP 2019 ESC guidelines for the diagnosis and management of acute pulmonary embolism developed in collaboration with the European respiratory society (ERS): the task force for the diagnosis and management of acute pulmonary embolism of the European society of cardiology (ESC). *Eur Heart J.* (2019) 54:1901647. 10.1183/13993003.01647-2019 31473594

[15] Ortel TL Neumann I Ageno W Beyth R Clark NP Cuker A American society of hematology 2020 guidelines for management of venous thromboembolism: treatment of deep vein thrombosis and pulmonary embolism. *Blood Adv.* (2020) 4:4693–738. 10.1182/bloodadvances.2020001830 33007077 PMC7556153

[16] Agnelli G Buller HR Cohen A Curto M Gallus AS Johnson M Oral apixaban for the treatment of acute venous thromboembolism. *N Engl J Med.* (2013) 369:799–808. 23808982 10.1056/NEJMoa1302507

[17] Bauersachs R Berkowitz SD Brenner B Buller HR Decousus H Gallus AS Oral rivaroxaban for symptomatic venous thromboembolism. *N Engl J Med.* (2010) 363:2499–510. 10.1056/nejmoa1007903 21128814

[18] Buller HR Prins MH Lensin AW Decousus H Jacobson BF Minar E Oral rivaroxaban for the treatment of symptomatic pulmonary embolism. *N Engl J Med.* (2012) 366:1287–97. 10.1056/nejmoa1113572 22449293

[19] Hokusai-VTE Investigators, Büller HR Décousus H Grosso MA Mercuri M Middeldorp S Edoxaban versus warfarin for the treatment of symptomatic venous thromboembolism. *N Engl J Med.* (2013) 369:1406–15. 10.1056/nejmoa1306638 23991658

[20] Schulman S Kearon C Kakkar AK Mismetti P Schellong S Eriksson H Dabigatran versus warfarin in the treatment of acute venous thromboembolism. *N Engl J Med.* (2009) 361:2342–52. 10.1056/nejmoa0906598 19966341

[21] Schulman S Kakkar AK Goldhaber SZ Schellong S Eriksson H Mismetti P Treatment of acute venous thromboembolism with dabigatran or warfarin and pooled analysis. *Circulation.* (2014) 129:764–72. 10.1161/CIRCULATIONAHA.113.004450 24344086

[22] Chai-Adisaksopha C Crowther M Isayama T Lim W. The impact of bleeding complications in patients receiving target-specific oral anticoagulants: a systematic review and meta-analysis. *Blood.* (2014) 124:2450–8. 10.1182/blood-2014-07-590323 25150296

[23] Hirschl M Kundi M. Safety and efficacy of direct acting oral anticoagulants and vitamin K antagonists in non-valvular atrial fibrillation – a network meta-analysis of real-world data. *Vasa.* (2019) 48:134–47. 10.1097/MD.0000000000026883 30376416

[24] Abraham NS Noseworthy PA Yao X Sangaralingham LR Shah ND. Gastrointestinal Safety of direct oral anticoagulants: a large population-based study. *Gastroenterology.* (2017) 152: 1014–1022.e1. 10.1053/j.gastro.2016.12.018 28043907

[25] Barra ME Fanikos J Connors JM Sylvester KW Piazza G Goldhaber SZ. Evaluation of dose-reduced direct oral anticoagulant therapy. *Am J Med.* (2016) 129:1198–204. 10.1016/j.amjmed.2016.05.041 27341955

[26] Pollack CV Reilly PA Eikelboom J Glund S Verhamme P Bernstein RA Idarucizumab for dabigatran REVERSAL. *N Engl J Med.* (2015) 373:511–20. 26095746 10.1056/NEJMoa1502000

[27] Connolly SJ Crowther M Eikelboom JW Gibson CM Curnutte JT Lawrence JH Full study report of andexanet ALFA for bleeding associated with factor XA inhibitors. *N Engl J Med.* (2019) 380:1326–35. 10.1056/NEJMoa1814051 30730782 PMC6699827

[28] Steinberg BA Gao H Shrader P Pieper K Thomas L Camm AJ International trends in clinical characteristics and oral anticoagulation treatment for patients with atrial fibrillation: results from the GARFIELD-AF, ORBIT-AF I, and ORBIT-AF II registries. *Am Heart J.* (2017) 194:132–40. 10.1016/j.ahj.2017.08.011 29223431

[29] Sanghai S Wong C Wang Z Clive P Tran W Waring M Rates of potentially inappropriate dosing of direct-acting oral anticoagulants and associations with geriatric conditions among older patients with atrial fibrillation: the SAGE-AF study. *J Am Heart Assoc.* (2020) 9:e014108. 10.1161/JAHA.119.014108 32146898 PMC7335533

[30] Nopp S Ay C. Bleeding risk assessment in patients with venous thromboembolism. *Hamostaseologie.* (2021) 41:267–74. 10.1055/a-1339-9987 33626580

[31] Eikelboom JW Connolly SJ Brueckmann M Granger CB Kappetein AP Mack MJ Dabigatran versus warfarin in patients with mechanical heart valves. *N Engl J Med.* (2013) 369:1206–14. 23991661 10.1056/NEJMoa1300615

[32] Olesen JB Lip GYH Kamper AL Hommel K Køber L Lane DA Stroke and bleeding in atrial fibrillation with chronic kidney disease. *N Engl J Med.* (2012) 367:625–35. 22894575 10.1056/NEJMoa1105594

[33] Kuno T Takagi H Ando T Sugiyama T Miyashita S Valentin N Oral anticoagulation for patients with atrial fibrillation on long-term dialysis. *J Am Coll Cardiol.* (2020) 75:273–85. 31976865 10.1016/j.jacc.2019.10.059

[34] Königsbrügge O Meisel H Beyer A Schmaldienst S Klauser-Braun R Lorenz M Anticoagulation use and the risk of stroke and major bleeding in patients on hemodialysis: from the VIVALDI, a population-based prospective cohort study. *J Thromb Haemost.* (2021) 19:2984–96. 10.1111/jth.15508 34418291

[35] Pengo V Denas G Zoppellaro G Jose SP Hoxha A Ruffatti A Rivaroxaban VS warfarin in high-risk patients with antiphospholipid syndrome. *Blood.* (2018) 132:1365–71. 10.1182/blood-2018-04-848333 30002145

[36] Woller SC Stevens SM Kaplan D Wang TF Branch DW Groat D Apixaban compared with warfarin to prevent thrombosis in thrombotic antiphospholipid syndrome: a randomized trial. *Blood Adv.* (2022) 6:1661–70. 10.1182/bloodadvances.2021005808 34662890 PMC8941474

[37] Mackman N. Triggers, targets and treatments for thrombosis. *Nature.* (2008) 451:914–8. 10.1038/nature06797 18288180 PMC2848509

[38] Weitz JI Hirsh J. New anticoagulant drugs. *Chest.* (2001) 119:95s–107s. 11157644 10.1378/chest.119.1_suppl.95s

[39] Colman RW. Are hemostasis and thrombosis two sides of the same coin? *J Exp Med.* (2006) 203:493–5. 10.1084/jem.20060217 16533890 PMC2118234

[40] van Montfoort ML Meijers JC. Anticoagulation beyond direct thrombin and factor XA inhibitors: indications for targeting the intrinsic pathway? *Thromb Haemost.* (2013) 110:223–32. 10.1160/TH12-11-0803 23739841

[41] Smith SA Travers RJ Morrissey JH. How it all starts: initiation of the clotting cascade. *Crit Rev Biochem Mol Biol.* (2015) 50:326–36. 10.3109/10409238.2015.1050550 26018600 PMC4826570

[42] Hoffman M Monroe DM III. A cell-based model of hemostasis. *Thromb Haemost.* (2001) 85:958–65. 10.1055/s-0037-1615947 11434702

[43] Ho KM Pavey W. Applying the cell-based coagulation model in the management of critical bleeding. *Anaesth Intensive Care.* (2017) 45:166–76. 10.1177/0310057X1704500206 28267938

[44] Zaidi A Green L. Physiology of haemostasis. *Anaesth Intensive Care Med.* (2019) 20:152–8. 1932177

[45] Renné T Schmaier AH Nickel KF Blombäck M Maas C. *In vivo* roles of factor XII. *Blood.* (2012) 120:4296–303. 10.1182/blood-2012-07-292094 22993391 PMC3507141

[46] Kravtsov DV Matafonov A Tucker EI Sun MF Walsh PN Gruber A Factor XI contributes to thrombin generation in the absence of factor XII. *Blood.* (2009) 114:452–8. 10.1182/blood-2009-02-203604 19351955 PMC2714215

[47] Lin L Wu M Zhao J. The initiation and effects of plasma contact activation: an overview. *Int J Hematol.* (2017) 105:235–43. 10.1007/s12185-016-2132-x 27848184

[48] Björkqvist J Nickel KF Stavrou E Renné T. *In vivo* activation and functions of the protease factor XII. *Thromb Haemost.* (2014) 112:868–75. 10.1160/th14-04-0311 25187064

[49] Schmaier AH. The contact activation and kallikrein/kinin systems: pathophysiologic and physiologic activities. *J Thromb Haemost.* (2016) 14:28–39. 10.1111/jth.13194 26565070

[50] Long AT Kenne E Jung R Fuchs TA Renné T. Contact system revisited: an interface between inflammation, coagulation, and innate immunity. *J Thromb Haemost.* (2016) 14:427–37. 10.1111/jth.13235 26707513

[51] Oehmcke-Hecht S Köhler J. Interaction of the human contact system with pathogens—an update. *Front Immunol.* (2018) 9:312. 10.3389/fimmu.2018.00312 29535715 PMC5834483

[52] Badimon L Vilahur G. Thrombosis formation on atherosclerotic lesions and plaque rupture. *J Intern Med.* (2014) 276:618–32. 10.1111/joim.12296 25156650

[53] Plow EF Wang Y Simon DI. The search for new antithrombotic mechanisms and therapies that may spare hemostasis. *Blood.* (2018) 131:1899–902. 10.1182/blood-2017-10-784074 29467183 PMC5921961

[54] Schmaier AH McCrae KR. The plasma kallikrein-kinin system: its evolution from contact activation. *J Thromb Haemost.* (2007) 5:2323–9. 10.1111/j.1538-7836.2007.02770.x 17883591

[55] Schmaier AH. A Novel antithrombotic mechanism mediated by the receptors of the kallikrein/kinin and renin–angiotensin systems. *Front Med.* (2016) 3:61. 10.3389/fmed.2016.00061 27965959 PMC5124569

[56] Weitz JI. Factor XI and factor XII as targets for new anticoagulants. *Thromb Res.* (2016) 141:S40–5. 10.1016/S0049-3848(16)30363-2 27207423

[57] Müller F Gailani D Renné T. Factor XI and XII as antithrombotic targets. *Curr Opin Hematol.* (2011) 18:349–55. 10.1097/MOH.0b013e3283497e61 21730835 PMC4364027

[58] Puy C Rigg RA McCarty OJT. The hemostatic role of factor XI. *Thromb Res.* (2016) 141 (Suppl. 2):S8–11. 10.1016/S0049-3848(16)30354-1 27207433 PMC6135087

[59] Weitz JI Chan NC. Advances in antithrombotic therapy. *Arterioscler Thromb Vasc Biol.* (2019) 39:7–12. 30580558 10.1161/ATVBAHA.118.310960

[60] Key NS. Epidemiologic and clinical data linking factors XI and XII to thrombosis. *Hematol Am Soc Hematol Educ Program.* (2014) 2014:66–70. 10.1182/asheducation-2014.1.66 25696836

[61] Gill D Georgakis MK Laffan M Sabater-Lleal M Malik R Tzoulaki I Genetically determined FXI (Factor XI) levels and risk of stroke. *Stroke.* (2018) 49:2761–3. 10.1161/STROKEAHA.118.022792 30355187

[62] Yuan S Burgess S Laffan M Mason AM Dichgans M Gill D Genetically proxied inhibition of coagulation factors and risk of cardiovascular disease: a mendelian randomization study. *J Am Heart Assoc.* (2021) 10:e019644. 10.1161/JAHA.120.019644 33834859 PMC8174173

[63] Preis M Hirsch J Kotler A Zoabi A Stein N Rennert G Factor XI deficiency is associated with lower risk for cardiovascular and venous thromboembolism events. *Blood.* (2017) 129:1210–5. 10.1182/blood-2016-09-742262 28039189

[64] Salomon O Steinberg DM Koren-Morag N Tanne D Seligsohn U. Reduced incidence of ischemic stroke in patients with severe factor XI deficiency. *Blood.* (2008) 111:4113–7. 10.1182/blood-2007-10-120139 18268095

[65] Salomon O Steinberg DM Zucker M Varon D Zivelin A Seligsohn U. Patients with severe factor XI deficiency have a reduced incidence of deep-vein thrombosis. *Thromb Haemost.* (2011) 105:269–73. 10.1160/TH10-05-0307 21057700

[66] Duga S Salomon O. Congenital factor XI deficiency: an update. *Semin Thromb Hemost.* (2013) 39:621–31. 10.1055/s-0033-1353420 23929304

[67] Peyvandi F Palla R Menegatti M Siboni SM Halimeh S Faeser B Coagulation factor activity and clinical bleeding severity in rare bleeding disorders: results from the European network of rare bleeding disorders. *J Thromb Haemost.* (2012) 10:615–21. 10.1111/j.1538-7836.2012.04653.x 22321862

[68] Löwenberg EC Meijers JC Monia BP Levi M. Coagulation factor XI as a novel target for antithrombotic treatment. *J Thromb Haemost.* (2010) 8:2349–57. 10.1111/j.1538-7836.2010.04031.x 20727068

[69] Bolton-Maggs PH Patterson DA Wensley RT Tuddenham EG. Definition of the bleeding tendency in factor XI-deficient kindreds–a clinical and laboratory study. *Thromb Haemost.* (1995) 73:194–202. 10.1055/s-0038-1653750 7792729

[70] Santoro C Di Mauro R Baldacci E De Angelis F Abbruzzese R Barone F Bleeding phenotype and correlation with factor XI (FXI) activity in congenital FXI deficiency: results of a retrospective study from a single centre. *Haemophilia.* (2015) 21:496–501. 10.1111/hae.12628 25623511

[71] Bolton-Maggs PH. Factor XI deficiency—resolving the enigma? *Hematology.* (2009) 2009:97–105. 10.1182/asheducation-2009.1.97 20008187

[72] Lewandowska MD Connors JM. Factor XI deficiency. *Hematol Oncol Clin North Am.* (2021) 35:1157–69. 34535287 10.1016/j.hoc.2021.07.012

[73] Georgi B Mielke J Chaffin M Khera AV Gelis L Mundl H Leveraging human genetics to estimate clinical risk reductions achievable by inhibiting factor XI. *Stroke.* (2019) 50:3004–12. 10.1161/STROKEAHA.119.026545 31558144 PMC6824502

[74] Wang X Smith PL Hsu MY Gailani D Schumacher WA Ogletree ML Effects of factor XI deficiency on ferric chloride-induced vena cava thrombosis in mice. *J Thromb Haemost.* (2006) 4:1982–8. 10.1111/j.1538-7836.2006.02093.x 16961605

[75] Wang X Cheng Q Xu L Feuerstein GZ Hsu MY Smith PL Effects of factor IX or factor XI deficiency on ferric chloride-induced carotid artery occlusion in mice. *J Thromb Haemost.* (2005) 3:695–702. 10.1111/j.1538-7836.2005.01236.x 15733058

[76] Ay C Hisada Y Cooley BC Mackman N. Factor XI-deficient mice exhibit increased bleeding after injury to the saphenous vein. *J Thromb Haemost.* (2017) 15:1829–33. 10.1111/jth.13766 28677246

[77] Schumacher WA Seiler SE Steinbacher TE Stewart AB Bostwick JS Hartl KS Antithrombotic and hemostatic effects of a small molecule factor XIA inhibitor in rats. *Eur J Pharmacol.* (2007) 570:167–74. 10.1016/j.ejphar.2007.05.043 17597608

[78] Lin J Deng H Jin L Pandey P Quinn J Cantin S Design, synthesis, and biological evaluation of peptidomimetic inhibitors of factor XIA as novel anticoagulants. *J Med Chem.* (2006) 49:7781–91. 10.1021/jm060978s 17181160

[79] Gailani D Renné T. The intrinsic pathway of coagulation: a target for treating thromboembolic disease? *J Thromb Haemost.* (2007) 5:1106–12. 10.1111/j.1538-7836.2007.02446.x 17388803

[80] Weitz JI Fredenburgh JC. Factors XI and XII as targets for new anticoagulants. *Front Med.* (2017) 4:19. 10.3389/fmed.2017.00019 28286749 PMC5323386

[81] Younis HS Crosby J Huh JI Lee HS Rime S Monia B Antisense inhibition of coagulation factor XI prolongs APTT without increased bleeding risk in cynomolgus monkeys. *Blood.* (2012) 119:2401–8. 10.1182/blood-2011-10-387134 22246038

[82] Matafonov A Leung PY Gailani AE Grach SL Puy C Cheng Q Factor XII inhibition reduces thrombus formation in a primate thrombosis model. *Blood.* (2014) 123:1739–46. 10.1182/blood-2013-04-499111 24408325 PMC3954054

[83] Gruber A Hanson SR. Factor XI-dependence of surface- and tissue factor-initiated thrombus propagation in primates. *Blood.* (2003) 102:953–5. 10.1182/blood-2003-01-0324 12689935

[84] Tucker EI Marzec UM White TC Hurst S Rugonyi S McCarty OJ Prevention of vascular graft occlusion and thrombus-associated thrombin generation by inhibition of factor XI. *Blood.* (2009) 113:936–44. 10.1182/blood-2008-06-163675 18945968 PMC2630279

[85] Büller HR Bethune C Bhanot S Gailani D Monia BP Raskob GE Factor XI antisense oligonucleotide for prevention of venous thrombosis. *N Engl J Med.* (2014) 372:232–40. 10.1056/NEJMoa1405760 25482425 PMC4367537

[86] Weitz JI Bauersachs R Becker B Berkowitz SD Freitas MCS Lassen MR Effect of osocimab in preventing venous thromboembolism among patients undergoing knee arthroplasty: the FOXTROT randomized clinical trial. *JAMA.* (2020) 323:130–9. 10.1001/jama.2019.20687 31935028 PMC6990695

[87] Verhamme P Yi BA Segers A Salter J Bloomfield D Büller HR Abelacimab for prevention of venous thromboembolism. *N Engl J Med.* (2021) 385:609–17. 10.1056/nejmoa2105872 34297496

[88] Weitz JI Strony J Ageno W Gailani D Hylek EM Lassen MR Milvexian for the prevention of venous thromboembolism. *N Engl J Med.* (2021) 385:2161–72. 10.1056/NEJMoa2113194 34780683 PMC9540352

[89] Walsh M Bethune C Smyth A Tyrwhitt J Jung SW Yu RZ Phase 2 study of the factor XI antisense inhibitor IONIS-FXI(Rx) in patients with ESRD. *Kidney Int Rep.* (2022) 7:200–9. 10.1016/j.ekir.2021.11.011 35155859 PMC8820988

[90] Lorentz CU Tucker EI Verbout NG Shatzel JJ Olson SR Markway BD The contact activation inhibitor AB023 in heparin-free hemodialysis: results of a randomized phase 2 clinical trial. *Blood.* (2021) 138:2173–84. 10.1182/blood.2021011725 34086880 PMC8641100

[91] Koch AW Schiering N Melkko S Ewert S Salter J Zhang Y MAA868, a novel FXI antibody with a unique binding mode, shows durable effects on markers of anticoagulation in humans. *Blood.* (2019) 133:1507–16. 10.1182/blood-2018-10-880849 30692123

[92] Lorentz CU Verbout NG Wallisch M Hagen MW Shatzel JJ Olson SR Contact activation inhibitor and factor XI antibody, AB023, produces safe, dose-dependent anticoagulation in a phase 1 first-in-human trial. *Arterioscler Thromb Vasc Biol.* (2019) 39:799–809. 10.1161/ATVBAHA.118.312328 30700130 PMC6494446

[93] Chan NC Weitz JI. AB023, A novel antibody that binds factor XI and blocks its activation by factor XIIa. *Arterioscler Thromb Vasc Biol.* (2019) 39:533–5. 10.1161/ATVBAHA.119.312459 30917049

[94] Kubitza D Heckmann M Distler J Koechel A Schwers S Kanefendt F. Pharmacokinetics, pharmacodynamics and safety of BAY 2433334, a novel activated factor XI inhibitor, in healthy volunteers: a randomized phase 1 multiple-dose study. *Br J Clin Pharmacol.* (2022) 1–16. 10.1111/bcp.15230 35014061 PMC9311154

[95] Piccini JP Caso V Connolly SJ Fox KAA Oldgren J Jones WS Safety of the oral factor Xia inhibitor asundexian compared with apixaban in patients with atrial fibrillation (PACIFIC-AF): a multicentre, randomised, double-blind, double-dummy, dose-finding phase 2 study. *Lancet.* (2022) 399:1383–90. 10.1016/S0140-6736(22)00456-1 35385695

